# *Streptophyta* and Acetic Acid Bacteria Succession Promoted by Brass in Slow Sand Filter System *Schmutzdeckes*

**DOI:** 10.1038/s41598-019-43489-9

**Published:** 2019-05-07

**Authors:** Ma. Carmen E. Delgado-Gardea, Patricia Tamez-Guerra, Ricardo Gomez-Flores, Mariela Garfio-Aguirre, Beatriz A. Rocha-Gutiérrez, César I. Romo-Sáenz, Francisco Javier Zavala-Díaz de la Serna, Gilberto Eroza-de la Vega, Blanca Sánchez-Ramírez, María del Carmen González-Horta, María del Rocío Infante-Ramírez

**Affiliations:** 1 Universidad Autónoma de Nuevo León, Facultad de Ciencias Biológicas, Departamento de Microbiología e Inmunología, Ave. Universidad s/n, San Nicolás de los Garza, N.L. 66450 Mexico; 2 Universidad Autónoma de Chihuahua, Laboratorio de Biotecnología, Facultad de Ciencias Químicas, Circuito Nuevo Campus Universitario No. 1, Chihuahua, 31125 Mexico

**Keywords:** Environmental impact, Biofilms

## Abstract

Macro- and microorganism activities are important for the effectiveness of the slow sand filtration (SSF), where native microorganisms remove contaminants mainly by substrate competition, predation, and antagonism. The aim of the present study was to evaluate the addition of the oligodynamic metals iron, copper, and brass, inserted separately into SSF to enhance pollutant removal in water samples. Four laboratory-scale SSFs were built and tested: control, iron, copper, and brass. Water analysis included physicochemical evaluation, total and fecal coliform quantification. An analysis on microbial communities in the SSFs *schmutzdecke* was achieved by using 16S rRNA amplification, the Illumina MiSeq platform, and the QIIME bioinformatics software. The results demonstrated that inorganic and organic contaminants such as coliforms were removed up to 90%. The addition of metals had no significant effect (*p* > 0.05) on the other parameters. The microbial community analysis demonstrated different compositions of the SSF with brass-influent, where the eukaryote *Streptophyta* was predominant (31.4%), followed by the acetic acid bacteria *Gluconobacter* (24.6%), and *Acetobacteraceae* (7.7%), these genera were absent in the other SSF treatments. In conclusion, the use of a SSF system can be a low cost alternative to reduce microbial contamination in water and thus reduce gastrointestinal diseases in rural areas.

## Introduction

Water resources are a major concern, and at least 2.3 billion people in the world drink contaminated water (WHO, 2015). Water pollutants have broad implications for environmental fluctuations and health economic resources. The slow sand filtration (SSF) system has become a useful tool in water potabilization processes, because it is easy to handle and has low operational costs with minimal energy and maintenance requirements^1^. SSF is a natural environmental process similar to the filtration of rain water passing through soil strata to aquifers and underground rivers; in this process microbiological pollutants are retained by soil^2^. SSF combines physical, biological, and chemical processes to obtain water free from particles and pathogens^3^.

SSF system operation is based on a mechanical filtration process that traps organic and inorganic contaminants in very small size grains, where native microorganisms use them as edible substrates. This process allows the biolayer, known as *schmutzdecke*, to provide a rich nutrient environment that promotes microbial growth and biofilm formation^4^. Once the microbial community, including coliforms, present in the filter reaches the maturation stage, it is effectively removed from the water^5^. In the SSF system, algae and bacterial predation, detritus compaction, microorganism death, and organic carbon reduction are the main biological mechanisms responsible for contaminant removal^6^.

The major organisms present in the *schmutzdecke* are algae, flagellates, ciliates, flat worms, rotifers, gastrotriches, nematodes, arthropods, and annelids. The predominant microorganisms are prokaryotes, microalgae, protozoa, and viruses. Among the prokaryotes, Gram negative bacteria in SSF are classified as oligotrophic, and some predominant bacteria are pigmented, such as *Pseudomonas* and *Aeromonas*^7^. In the SSF system, predation is an important factor that allows enteric microorganism removal during water filtration. Algae species like *Chrysophyte* are known as a bacteria predators and may produce antibacterial toxins that help to reduce the coliform count^8^. Elucidation of SSF-colonizing microorganisms by metagenomics analysis is essential to improve the understanding of the operational conditions needed to improve this system^9^. It has been demonstrated that heavy metals like iron, copper, and brass have antimicrobial activity due to the oligodynamic effect, which may occur at low concentrations of these metals. When bacterial cells are exposed to heavy metals, metallic ions are delivered to the cell and bound to DNA, enzymes, and proteins, disrupting membrane permeability and causing cell death^10^, which facilitates microorganism removal. The aim of the present study was to evaluate the removal of inorganic and organic pollutants present in water collected from a dam and a river located near to Chihuahua city in Mexico after the addition of an oligodynamic metal—iron, copper, or brass—into a SSF system.

## Material and Methods

### Artificial water preparation

A mixture of 1% water from Sacramento river near to rural communities in the outskirts of the city (28°33′46.5″N 106°10′31.5″W) and 99% water from a dam that provides water to the city of Chihuahua (28°33′46.5″N 106°10′31.5″W) was prepared to fill out the sand filters. The water from the dam and from the river was characterized to assure the formation of the s*chmutzdecke* in the SSF based on the microbiological composition and physicochemical parameters. This mixture was stored at 4 °C until it was used to feed the filters. Samples were collected from both water bodies following the Mexican environmental procedures (NOM-014-SSA1-1993). One hundred and sixty liters of water was collected weekly from the dam and 2 L was collected from the river every 15 days for 20 weeks^11^.

### Setting up the SSF systems

Granular sand from water sample sites was sifted and washed to remove clay and fine silt. The middle area of the filter was sifted with fine sand and filtered with an 0.45 mm pore-size, 20 cm diameter stainless iron sieve (Ibili, Guipúzcoa, Spain). Coarser sand was sifted with an 8″ size brass sieve with no. 20 mesh (0.85 mm; USA standard ASTM E-11). Filters were half-filled with tap water to prevent air bubble formation in the filter core^12^.

Four 5 cm columns were used for each gravel filter to cover the drainage entirely; coarse sand of 0.8 mm thickness was added up to a height of 5 cm^12^. Then, 0.4 mm fine sand was added up to a height 60 cm. Supernatant fluid was filled up to a height of 35 cm over the sand layer^13,14^.

Regarding the experimental design, 0.253 g of each metal, representing about 10% of the fine sand’s total volume, was evenly mixed over the top layers. Filters were marked as SSF-1 for the untreated control, SSF-2 for the filter with an iron stick, SSF-3 for the filter with copper, and SSF-4 for the filter with brass. An iron nail (2 cm length, 3 mm width) with 98% iron content was inserted into SSF-2. Copper (99.9% copper) was added to SSF-3 in a thick wire presentation using ~2 cm long pieces. Brass, mainly consisting of a copper and zinc alloy (68.5–71.5% copper; 0.07% lead; 0.05% iron; 28.38–31.38% zinc) was added to SSF-4 in a ~2 cm long cut sheet presentation^14^.

### Filtration system design

The supporting structure was built with a metal base 1.69 m in height, 1.30 m in length, and 0.40 m in width. Four filters were fastened to the metal base with metallic clamps. Each filter was built with PVC (polyvinyl chloride) transparent industrial hose with a height of 1.10 m and a diameter of 2″ (Supplementary Fig. S1). The filter outlet consisted of a half-inch ball valve connected to a quarter-inch needle valve. A 50-L capacity, high-density polyethylene (HDPE) food grade water jug was set up over the filters as a water feeder. The HDPE was filled with the river and dam water mixture by gravity, connected to the sand filter, and the valve was open for filters to be filled with the water mixture by gravity. The volume filtration rate was adjusted to 0.1 L/h at the lab scale (register in process/MX/E/2019/004622).

### Water analysis

Water samples were bacteriologically and physicochemistry analyzed before and after passing through the SSF. Bacteriological analyses of the raw water and the water after SSF treatment were performed by cleaning the filter output with sodium hypochlorite solution (100 mg/L). A raw water sample was collected at least 3 min after the final output. Sterile glass bottles were used to collect each sample from the filter for microbiological analysis. Filtered sampling collection was performed as indicated in the Mexican Standard Procedures (NOM-014-SSA1-1993). Water samples were characterized by determining physicochemical parameters—pH, turbidity, and total dissolved solids (SDT)—weekly^4,5^.

### Physicochemical analysis

TDS were measured in the laboratory immediately after sampling using a multi-parameter instrument (HI-98130 pH/CE/TDS/°C, HANNA Instruments, Woonsocket, RI, USA). The pH was determined in the laboratory using a pH meter (HI 2210, Hanna Instruments, Woonsocket, Rhode Island, USA). For both measures, the electrodes were properly rinsed with deionized water prior to every measurement to avoid sample contamination. The samples were stored on ice during transportation and then stored at 4 °C until analysis.

### Microbiological analysis

Influent and effluent microbiological analyses were carried out as indicated in the standard Mexican method “Water analysis—enumeration of organisms total coliforms, fecal coliform organisms (thermotolerant), and *Escherichia coli*—most probable number (MPN) method in multiple tubes” (NMX-AA-042-SCFI-2005 regulation).

### Total coliform removal efficiency

The total coliform removal efficiency was determined by calculating the bacterial removal percentage from total coliforms (BRTC) according to the Mexican Standard Procedures (NOM-244-SSA1-2008 regulation) using the following formula:

$$ \% \,{\boldsymbol{BRTC}}=\frac{{(totalcoliforms)}_{1}-{(totalcoliforms)}_{2}}{{(totalcoliforms)}_{1}}\times 100$$

where % ***BRTC*** is the bacterial removal percentage from total coliforms, _1_. is the count of total coliform organisms in MPN/100 mL or CFU/100 mL of untreated test water, and _2_. is the count of total coliform organisms in MPN/100 mL or CFU/100 mL of tested water.

### *Schmutzdecke* sample collection

Sand samples were collected at week 16, once the filters were considered mature. For s*chmutzdecke* (biofilm) analysis, a 5 g (dry weight) sample was obtained from the first 5 cm above the fine sand from each filter with sterile metal crushing. Samples were then stored in 50 mL Falcon tubes at −20 °C until use^2^.

### DNA extraction

Biolayer (*schmutzdecke*) samples were mixed with 13.5 mL of DNA extraction buffer (100 mM Tris-HCl; pH 8.0; USB, Cleveland, Ohio), 100 mM sodium EDTA (pH 8.0; Laboratorios LAITZ S.A., México, D.F.), 100 mM sodium phosphate (pH 8.0), 1.5 M NaCl, and 1% CTAB (Sigma-Aldrich Química, S.L., Toluca, México), and 50 μL proteinase K (10 mg/mL; Invitrogen™, Carlsbad, CA). Falcon plastic conic tubes were stirred at 225 rpm for 30 min at 37 °C. Next, 1.5 mL SDS (20%) was added into the mix and incubated in a water bath at 65 °C for 2 h, with the tubes gently inverted every 15 min. The, the tubes were centrifuged at 6000 g for 10 min at room temperature.

Supernatants were transferred to a cleaned 50 mL Falcon tube. Next, pellets were extracted twice with 4.5 mL extraction buffer and 0.5 mL SDS (20%). For this, a vortex was used for 10 s, samples were incubated in a water bath at 65 °C during 10 min, and tubes were centrifuged as mentioned above. All supernatants were combined and mixed with an equal volume of chloroform–isoamyl alcohol (24:1, Sigma-Aldrich Química, S.L.). The aqueous phase was recovered by centrifugation and precipitation with an 0.6 volume of isopropanol overnight at −20 °C, after which nucleic acids were obtained by centrifugation at 16,000 × g for 3 min at room temperature.

The final pellet was washed with 70% cold ethanol (Jalmek Científica S.A. de C.V., San Nicolás de los Garza, N.L., MX) and suspended in sterile deionized water to a final volume of 200 µL^2^. Extracted DNA was analyzed in 1% agarose gel electrophoresis and visualized with UV transiluminator UV GelLogic 200 (Eastman Kodak Company, NY). For data recording, the KODAK 1D 3.6 software program was used.

### DNA purification and quantification and sequencing

DNA samples were purified with Zymoclean™ Gel DNA Recovery Kit (Control Técnico y Representaciones, S.A. de C.V., Monterrey, N.L.) according to the manufacturer’s instructions. Purified samples were quantified with the Nanodrop ND-1000-UV-Vis spectrophotometer (Nanodrop Technologies, Wilmington, DE).

Purified DNA samples were sent to Macrogen Inc. (Macrogen Inc., Seoul, Rep. De Korea), for “*paired-end”* amplification of the 16S rRNA bacterial gene through the Illumina MiSeq platform (San Diego, CA).

Library construction was carried out by DNA sample aleatory fragmentation and 5′ and 3′ adapter ligation. Fragments linked to the adapter were amplified by PCR, and the gel was purified.

For sequence cluster generation, the library was loaded into a flow cell where the fragments were captured in a lawn of oligonucleotides attached to the surface, which were complementary to the library adapters. Next, each fragment was amplified in different clonal clusters through bridge amplification. When the generation of clusters was complete, templates were analyzed by sequencing. All data were recorded after eliminating all incomplete and/or flawless sequences.

Sequenced data were converted to unfiltered data for analysis. Illumina’s Miseq platform generates raw images using MCS Software (MiSeq Control Software v2.2) for system control through integrated primary analysis software called RTA (Real Time Analysis, v1.18, Illumina). BCL binary information (base calls) was converted to FASTQ using the Illumina BCL2FASTQ package (v.8.4).

### Microbial sequences analysis

Obtained sequences were analyzed with the QIIME bioinformatics program (Quantitative Insights into Microbial Ecology v.1.9.1-20150604. Once the sequences had been obtained, the bioinformatics analysis was carried out by following the tutorial shown by QIIME software (version 1.9.1).

### Statistical analysis

For physicochemical analysis, one-way ANOVA was performed to determine significant differences among SSF treatments (SPSS Inc., Chicago, IL).

Total and fecal coliform data were transformed into Log^10^ to normalize data. Analysis of variance (ANOVA) was applied to detect statistically differences among SSF treatments and the SSF control). To determine the influences of different factors when biofilm was sampled, a covariance analysis was performed. Data were analyzed with SPSS v.22 (SPSS Inc., Chicago, IL).

All data from the metagenomics analysis, physicochemical analysis, and MPN statistical analysis are available in attached documents.

## Results and Discussion

### Soil characterization

The soil physicochemical characterization results are shown in Supplementary Table S1. The texture results from SSF indicated that it was a sandy soil (A) with a sand percentage ≥85% and ≤15% of silt and clay, according to the Mexican Standard Procedures (NOM-021-SEMARNAT-2000). Sandy soil allows water to pass through the filter without being retained. Soils in which sand or gravel predominates have good drainage aeration and do not offer resistance to tillage, which benefits microorganisms’ activity^7^. Sandy soils with an apparent density texture between 1.2–1.3 g/mL are preferred for SSF systems, because sand particles are in intimate contact due to the low organic matter content^13^.

### Effluent and influent analysis

The microbiological analysis revealed that the Sacramento river contains a high level of microorganisms, and this sample helped to accelerate the ripening time for s*chmutzdecke* formation in the SSF. The results of the pH, turbidity, total dissolved solids, and percentage of bacterial and total coliform reduction are shown in Supplementary Fig. S2. SSF influent from the different tested systems showed a slight non-significant (*p* > 0.05) increase in pH value (Supplementary Fig. S2A) among treatments, where all recorded pH values (pH 6.6–8.5) remained under the Mexican Standard quality control given value (NOM-127-SSA1-1994 regulation).

The turbidity removal percentage showed a greater but non-significant (*p* > 0.05) removal percentage in filters where metals were added (Supplementary Fig. S2B). This may be attributed to the physical properties of the metals, since they can link to sulphates in water, causing both flocculation and coagulation^15^. The turbidity decrease was also associated with biofilm formation on the surface or within sand grains, catching solid particles in water^16,17^. Turbidity values in the SSF effluent were reported below the maximum level established in the Mexican Standard regulations for drinkingwater. Nevertheless, other studies using SSF systems reported higher values removal percentages of turbidity than in any of the treatments using in this study with a maximum of around 45%; Pfannes *et al*.^4^ reported >70%, Elliott *et al*.^16^ reported 74.9%, and Bagundol *et al*.^17^ reported 99.9%. The lower effectiveness in reducing turbidity could be a consequence of the bigger sand grains used in this study (0.45 mm); thus, the use of smaller-sized sand grains may improve particle retention in SSF systems.

Data obtained regarding total dissolved solids (TDS) showed no significant differences among treatments (*p* > 0.05) (Supplementary Fig. S2C). The results showed greater TDS removal in the control treatment (SSF-1), compared with that of the influent of metal-integrated SSF treatments. This higher TDS value can be associated with microorganism establishment within the *schmutzdecke*, which disintegrates cellular forming material assimilated by other microorganisms and eventually converts it into inorganic matter like carbon dioxide, nitrates, phosphates, and other similar salts^13^. Regardless of the TDS increase, values did not exceed the standard values for organoleptic property approval (500 mg/L) given by the Mexican standards^18^.

There were no differences in bacterial reduction of the total coliform organism percentage (BRCT) between the untreated control and SSF treatment groups (*p* > 0.05) (Supplementary Fig. S2D). The average BRCT was 96.8% in the untreated control (SSF-1), 95.05% with iron addition (SSF-2); 95.9% with copper addition (SSF-3), and 93.7% with brass addition (SSF-4). Results were similar to those reported by others. For instance, Jenkins *et al*.^11^ reported a 98.5% removal efficiency, whereas D’Alessio *et al*.^19^ reported a microbial removal efficiency of over 99%.

### Microbiological analysis

**Figure 1:**
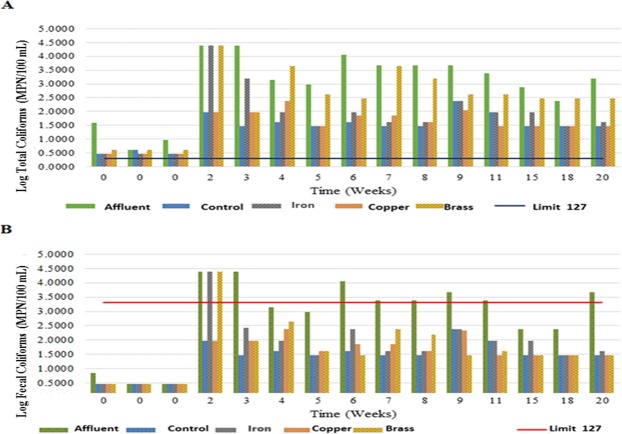
Most probable number (MPN) counts logarithm of (**A**) the total coliforms, where the blue line indicates the maximum limit according to the Mexican Standard procedures (NOM-127-SSA1-1994); (**B**) the fecal coliforms, where the red line indicates the maximum limit according to the Mexican Standard procedures (NOM-001-ECOL-1996). Results from the influent from SSF-1 (control); SSF-2 (iron); SSF-3 (copper); and SSF-4 (brass).

The bacterial counts were analyzed in the SSF tested systems from week 2 to week 20. MPN values are shown in Fig. 1. Despite BRCT percentage values being over 93.7% in all treatments (Fig. 1A), microbiological analyses indicated that the water effluent from SSF treatments cannot be used for human consumption. The coliform count surpassed Mexican Standard recommendations (NOM-127-SSA1-1994) in all SSF treatments, indicating an upper limit of 2 MPN/100 mL (0.3 log MPN/100 mL) total coliforms and no detectable fecal coliforms (Fig. 3A,B). The fecal coliform count was considerably lower from week 3 after filtration with all SSF treatments, based on the Mexican standard procedures range (NOM-001-ECOL-1996), which recommends a maximum permissible limit of 2000 MPN/100 mL or 3.3 log MPN/100 mL of pollutants in national water that receives residual water (Fig. 1B). Although the use of SSFs did not result in safe-to-drink water, the tested SSF systems represent a desirable approach for wastewater pretreatment before discharge into natural water bodies.

### Prokaryotic community identification

From the *schmutzdecke* samples taken from each SSF tested system, a total of 787,829 high-quality reads with a range from 140,000 to 265,000 were obtained, where the size of each prokaryotic amplified sequence was between 286 and 289 bp (Supplementary Table S2). Shannon and Simpson alpha index results showed a significant diversity supported by richness and species relative abundance, where Shannon values ranged from 6.45 to 11.03, and Simpson values ranged from 0.9942–0.9978 (Table 1). The prokaryote diversity index analysis indicated that *schmutzdecke* from the untreated control (SSF-1) and copper-added SSF-3 systems presented similar microbial diversity values, whereas the brass-added SSF-4 system showed a significantly (*p* < 0.05) lower diversity.

**Table 1:** Diversity indexes of the microbial community present in the slow sand filtration systems (SSFs) tested.

Sample ID	Shannon (H′)	Simpson (D)
SSF-1 (control)	10.22	0.9971
SSF-2 (iron)	11.03	0.9978
SSF-3 (copper)	10.49	0.9942
SSF-4 (brass)	6.45	0.9643

**Figure 2:**
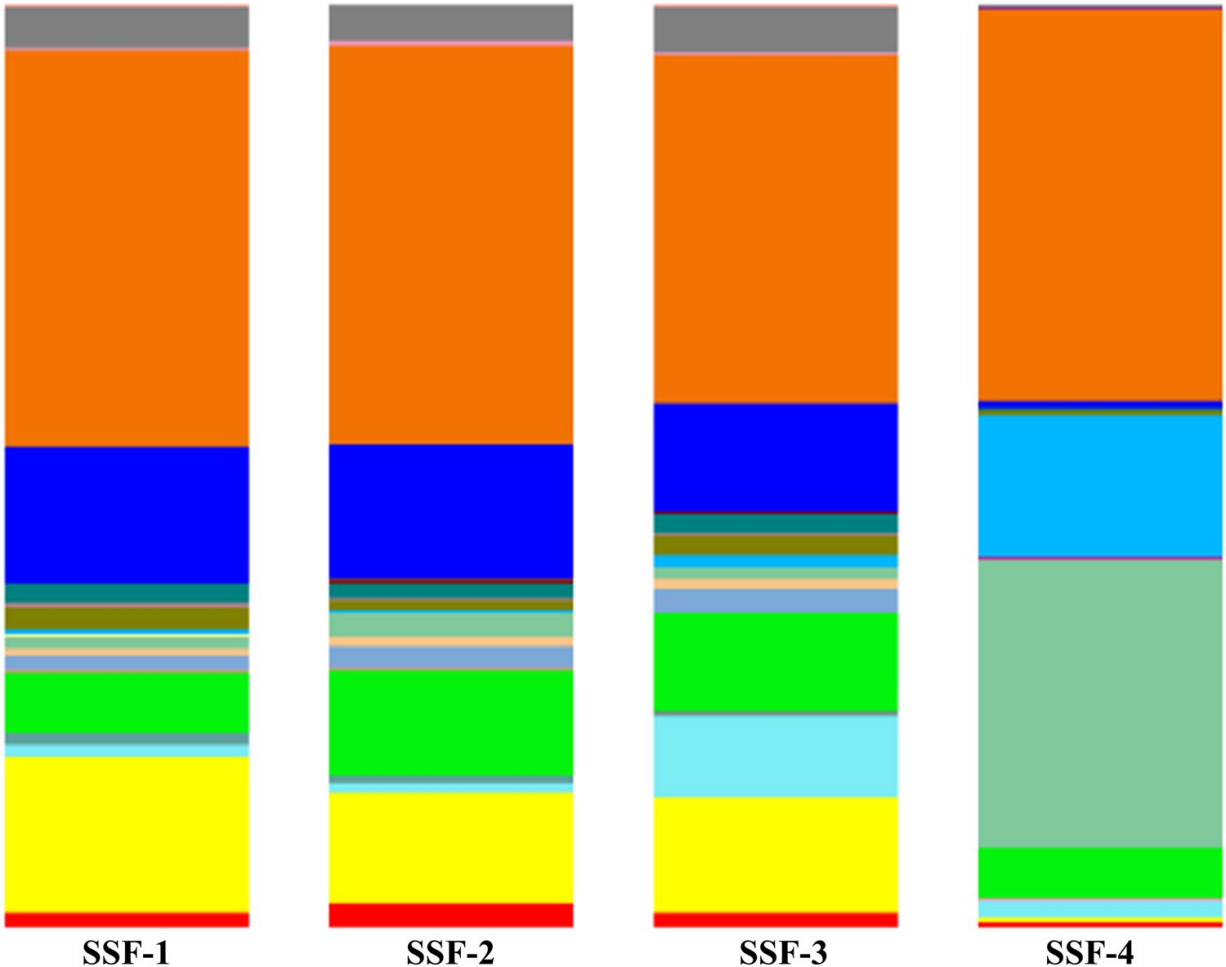
Dominant phyla in the tested slow sand filtration systems (SSFs). SSF-1 = control; SSF-2 = iron; SSF-3 = copper; SSF-4 = brass.

The dominant phylum in the *schmutzdecke* among SSF treatments was similar among SSF-1, SSF-2, and SSF-3 treatments; however, SSF-4 (brass-added) *schmutzdecke* presented significant (*p* < 0.05) differences in the prokaryotic community (Table 2, Fig. 2). Analysis revealed that the predominant phylum was Proteobacteria in all SSF treatments; this phylum has been reported as the predominant phylum in other studies^4,5,10,20^. Most species are Gram-negative with different metabolisms, including chemoorganotrophic, phototrophic, and chemo-lithotrophic species^7^.

**Table 2:** Dominant prokaryotic phyla percentages in the slow sand filtration systems (SSF) tested^1^.

Phyla	SSF-1 (control)	SSF-2 (iron)	SSF-3 (copper)	SSF-4 (brass)
Proteobacteria	*42.89%	*43.2%	*37.57%	*42.54%
Acidobacteria	*16.92%	*12.05%	*12.46%	0.4%
Planctomycetes	*14.9%	*14.64%	*11.74%	0.6%
Bacteroidetes	*6.46%	*11.39%	*10.64%	*5.46%
Verrucomicrobia	*4.38%	*3.83%	5.09%	0.3%
Actinobacteria	1.2%	0.9%	*8.69%	*1.9%
Firmicutes	0.7%	0.2%	1.2%	*15.5%
Cyanobacteria	1.3%	2.5%	1.2%	*31.1%

*The main five phyla detected in all the SSF *schmutzdeckes*. See Fig. 4 for their relative abundances.

^1^*Streptophyta* plant subdivision genera were detected.

Acidobacteria was the second most abundant phylum in the *schmutzdecke* in SSF tested systems, except for brass-added SSF-4; species of this phylum are soil habitats and may represent 52% of the total microbial community. There was a negative correlation between Acidobacteria and organic carbon concentration; this may be related to the oligotrophic capacity of this phylum’s members^21^.

The Actinobacteria phylum includes the actinomycetes class, a large group of filamentous soil bacteria, which are natural vegetable material and soil inhabitants^4,5,20^. The Planctomyces phylum was found in SSF; species of this phylum are mostly aerobic chemo-heterotrophs with hetero-polysaccharides degraders in lower numbers^22^.

Species from the Bacteroidetes phylum are normally associated with saccharolytic bacteria and may play roles in organic matter degradation^4^. The Verrucomicrobia phylum is represented by a few species found in aquatic marine and fresh water environments as well as in agricultural and forestry soils. Verrucomicrobia species are carbohydrate-fermenting aerobic bacteria, and some have symbiotic associations with protists^7^.

The Cyanobacteria phylum was the second most present in SSF-4 (brass-added). Cyanobacteria are oxygenic phototrophic bacteria; all species are CO_2_-fixing bacteria and some species fix dinitrogen. This finding is important since brass addition may favor Cyanobacteria establishment in secondary microbial successions in fresh water filtration systems. The Firmicutes phylum was the third most present phylum in the *schmutzdecke* of SSF systems, apart from SSF-4. This phylum is mostly represented by endospores-forming bacteria and lactic acid-producing bacteria^23^ (Table 2, Fig. 2). Data obtained in previous studies where microbial communities were characterized showed the presence of Proteobacteria, Nitrospirae, Acidobacteria, and Bacteroidetes with a steady biofilm in an activated sludge system for the water treatment process (Huang *et al*., 2017).

**Figure 3:**
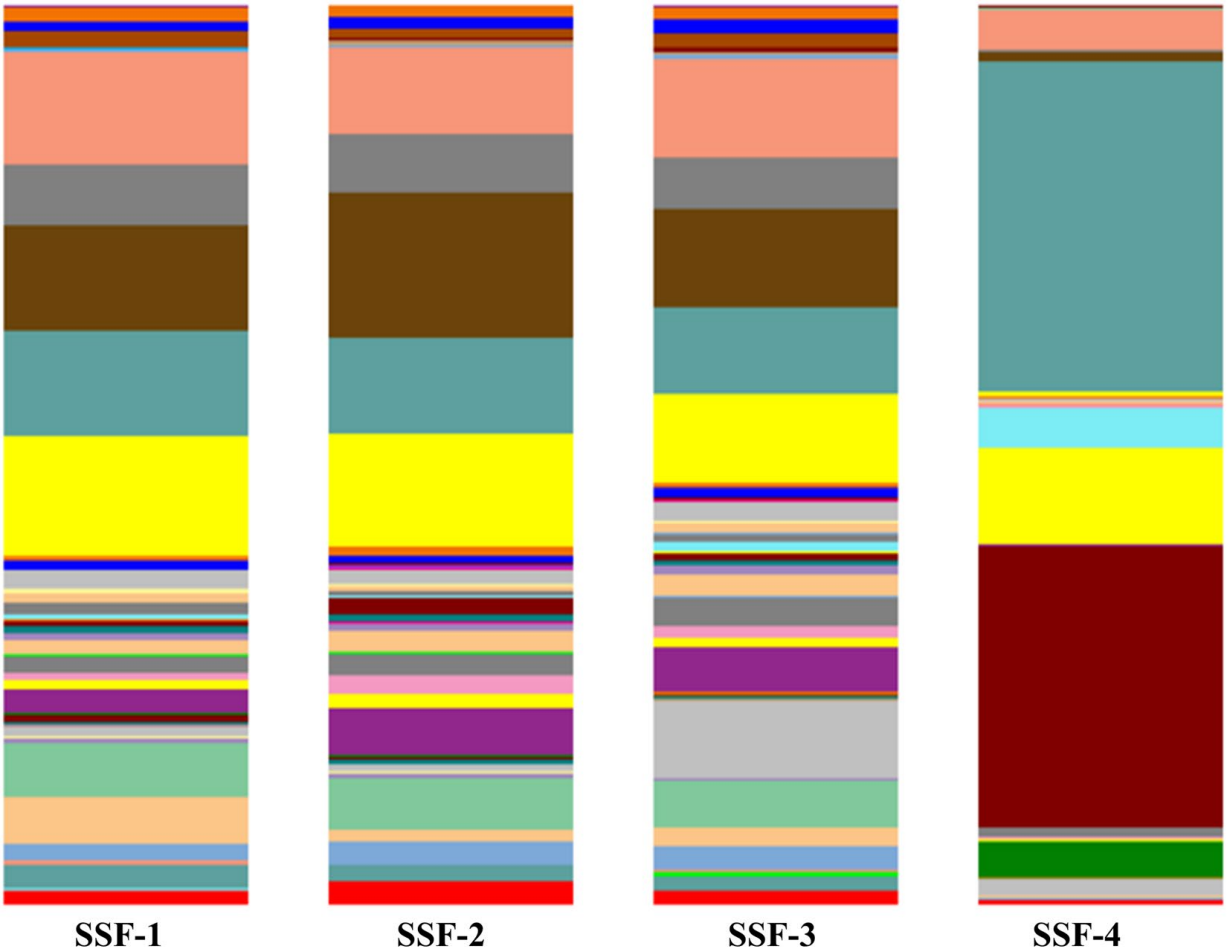
Dominant classes in the tested slow sand filtration systems (SSF). SSF-1 = control; SSF-2 = iron; SSF-3 = copper; SSF-4 = brass.

Similar to prokaryotic dominance analyses, the class analysis of *schmutzdecke* samples revealed differences between untreated and iron- and copper-added (SSF-1, SSF-2, and SSF-3) versus brass-added (SSF-4) treatments (Table 3, Fig. 3). The most abundant prokaryotic class detected in each *schmutzdecke* of the SSF tested systems was Alphaproteobacteria, and it was present at an extremely high level in the brass-added treatment (SSF-4) (Table 3, Fig. 3). Alphaproteobacteria habitually grow in low-nutrient concentration habitats^24^. Alphaproteobacteria, Betaproteobacteria, and Gammaproteobacteria classes belong to the Proteobacteria phylum. The Betaproteobacteria class has a variety of very important pathogens as well as methylotrophic and chemolithotrophic species^7^. Members of the Gammaproteobacteria class present an aerobic or fermenter (facultative) metabolism and are phototrophs, chemoorganotrophs, or chemolithotrophs^20^.

**Table 3:** Dominant prokaryotic classes in the tested slow sand filtration systems (SSF).

Phyla	Classes	SSF-1 (control)	SSF-2 (iron)	SSF-3 (copper)	SSF-4 (brass)
Proteobacteria	Alphaproteobacteria	*11.7%	*10.6%	*9.5%	*36.7%
	Betaproteobacteria	*11.7%	*16.2%	*11.1%	1.0%
	Gammaproteobacteria	*12.3%	*9.6%	*11.1%	*4.5%
Planctomycetes	Planctomycetia	*13.1%	*12.5%	*9.8%	0.4%
Acidobacteria	Chloracidobacteria	*6.0%	*5.8%	5.1%	0.0%
Bacteroidetes	Cytophagia	2.7%	5.3%	5.0%	0.0%
	Bacteroidia	0.1%	0.1%	0.1%	3.9%
Actinobacteria	Actinobacteria	1.0%	0.8%	*8.5%	0.9%
Cyanobacteria	Chloroplast	0.1%	0.0%	0.0%	*31.1%
Firmicutes	Bacilli	0.1%	0.0%	0.1%	*10.9%
	Clostridia	0.5%	0.1%	1.0%	*4.2%

*The five main classes were identified. *The main classes detected in all SSF *schmutzdeckes*.

The most abundant class detected in *schmutzdecke* from SSF-1, SSF-2, and SSF-3 was Planctomycetia (Table 3, Fig. 3). Planctomycetia includes heterotrophic and anaerobic ammonia-oxidizing bacteria like those from the *Anammox* genus. *Anammox* species oxidize ammonia into dinitrogen gas using nitrite as oxidizing agent under anaerobic conditions ^25^. *Chloracidobacterium*, a genus of the Acidobacteria class, includes chlorophyll-photosynthetic bacteria^26^. Cytophagia and Bacteroidia are classes that belong to the Bacteroidetes phylum. These classes comprise almost exclusively strictly aerobic species, although some species have limited fermentative metabolism. Some species degrade complex polysaccharides. Cytophagia species are well distributed in soils and fresh water environments, where they probably carry out cellulose digestion metabolism^27^.

The Actinobacteria class was present in SSF in a lower proportion (Table 3, Fig. 3). Actinobacteria species play critical roles in soil ecology, since the metabolism of several species includes dinitrogen fixation, phosphor solubilization, and mobilization of different nutrients. Actinobacteria species are well known by their ability to degrade recalcitrant polymers, like lignocellulose and chitin compounds, among others^28^.

Although the brass-added (SSF-4) *schmutzdecke* presented an extremely different microbial community, the inorganic and organic contaminant removal performance was not affected. In the SSF-4 *schmutzdecke*, Chloroplast, Bacilli, and Clostridia (4.2%) classes (Table 3, Fig. 3) were observed. Clostridia members are anaerobic bacteria, whereas Bacilli are strict aerobic or facultative species^23^.

**Figure 4:**
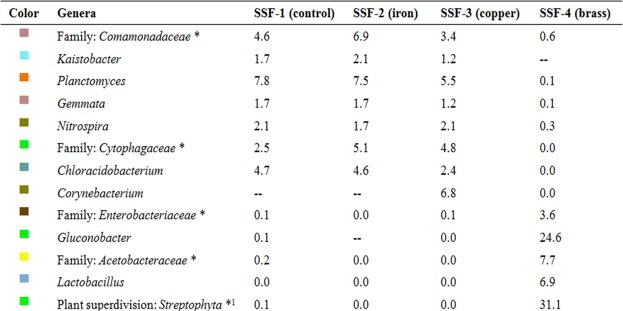
Dominant prokaryotic genera in the tested slow sand filtration systems (SSF). *Unknown genera. ^1^*Streptophyta* genera were recognized by the Illumina MiSeq platform and the QIIME bioinformatics program.

**Figure 5:**
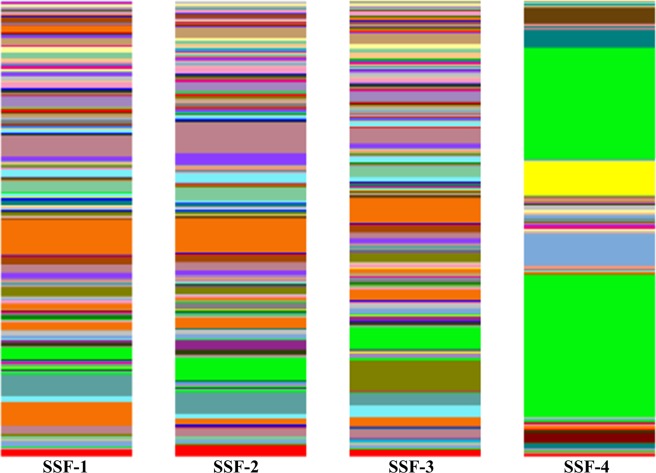
Dominant genera in the tested slow sand filtration systems (SSF). See Fig. 4 for detailed information. SSF-1 = control; SSF-2 = iron; SSF-3 = copper; SSF-4 = brass.

Regarding the dominant genera in the prokaryotic community, *Planctomyces* genus was predominant in the SSF-4 (brass-added) *schmutzdecke* (Figs 4 and 5). *Planctomyces* species are bacteria colonizing algae as they produce cellulose metabolic enzymes, which allows them to degrade carbohydrates present in plants and algae cell walls^9,22,29^. The *Comamonadaceae* (Betaproteobacteria class) genus belongs to a bacterial family that is responsible for denitrification and aromatic degradation processes^30–32^.

The *Chloracidobacteriun* genus from the *Acidobacteriaceae* family has been recently reported as a new genus^33^, which is related to organoheterotrophic bacteria. The *Cytophagaceae* (Cytophagia class) and *Gemmata* genera species are heterotrophic aerobic bacteria reported to live in marine and fresh water and terrestrial habitats^20^. *Gemmata* has a very slow growth rate (generation time of 11 h) and has a central role in the degradation of plants and algae, including other bacteria exopolysaccharides^22^. The *Kaistobacter* genus is distributed in terrestrial environments, and it is considered to include xenobiotic-degrader microorganisms (phenanthrene biodegradation metabolism)^34^. More than 50 *Corynebacterium* species have been recognized in soil, plant, and food habitats^35^ (Figs 4 and 5).

In the present study, the concentration of *Gluconobacter* was exceptionally high (24.6%) in the brass-added (SSF-4) *schmutzdecke*, whereas the *Streptophyta* (Eukaryota: Plantae) superdivision genera was detected in 31.1% of samples, being the most abundantly detected genus in SSF-4 (Figs 4 and 5).

The *Lactobacillus* genus was only detected in the brass-added *schmutzdecke*, being the third most abundantly detected genus (6.4%) in SSF-4. It is possible that microalgae stimulated the *Lactobacillus* abundance, since it has been reported that *Chlorella vulgaris* promotes *Lactobacillus* growth in synthetic culture medium^36^, whereas several lactic acid bacteria remove heavy metals from aqueous solutions^37^. In fact, *Lactobacillus* species remove lead and are resistant to zinc^38^.

Overall, the results indicated that the adsorption media in the SSF may not be the main factor in the pathogen eradication process. There may be factors for the erradication with higher influences like predation by protozoans and competitive nutrient and environmental conditions Pernthaler^39^. However, all of such mechanisms converged in an optimal performance of SSFs systems.

### Oligodynamic effect of brass in SSF

The antimicrobial actions, antibacterial in particular, which are exerted by certain metals in their elemental form, represent the oligodynamic effect^40^. In this study, brass addition to the SSF (SSF-4) resulted in an oligodynamic effect on *Acidobacteria*, *Planctomycetes*, and *Verrucomicrobia*. Zinc is an essential element for superior microorganisms, because it acts as a cofactor in many enzymatic reactions. A considerable increment of zinc concentration over the optimum level (10^−7^ M or 10^−5^ M depending on the bacterial strain) disturbs Zn^+2^ homeostasis and could be cytotoxic. This antibacterial activity may result from two mechanisms: (1) direct interaction with microbial membranes, leading to an increase in permeability and membrane destabilization, and (2) direct interaction with nucleic acids and enzyme deactivation^41^.

The microbial community in the SSF-4 was represented by acetic acid bacteria as *Gluconobacter* and *Acetobacteraceae* family members, *Streptophyta*, and *Lactobacillus*, which constitutes an important ecological application not just for contaminants removal, but for agriculture, food, clinical, and other highly valuable biotechnology production processes^42–45^.

The *Gluconobacter* and *Acetobacter* genera belong to the acetic acid bacteria group; they are strict aerobic bacteria and carry out incomplete oxidization of alcohol and carbohydrates through the pentose phosphate metabolic pathway, resulting in organic acid accumulation as the final product. The *Gluconobacter* metabolism products acetone and carbon dioxide can be used, respectively, by the *Acetobacteraceae* family and *Streptophyta* as the main carbon source to generate oxygen, which, in turn, stimulates *Gluconobacter* growth by oxidation activities^44^. Previous studies indicated that *Gluconobacter* solubilizes zinc (precipitated as zinc oxide salt), since it has a better ability to resist metals compared to other microbial species^46^. Similarly, several *Charophyta* alga species (eukaryotic) have shown high tolerance and capacity to accumulate heavy metals (including zinc resistance and lead bioaccumulation in high amounts) through calcium carbonate inlay co-precipitation^47^.

Although bacteria benefit from zinc, others, such as the *Planctomyces*, *Cytophagaceae* and *Comamonadaceae* genera, are sensitive to this metal^48^. Previous reports have indicated that zinc can diminish the richness and diversity of several families and species, whereas several heavy metals promote Proteobacteria populations and diminish Acidobacteria and Actinobacteria phyla^49^.

Another ecological approach to the brass’ oligodynamic effect is *Lactobacillus* genus selection thanks to the resistance to zinc of this genus. Indeed, *Lactobacillus* can remove heavy metals from aqueous solutions, thus favoring the establishment of other species over time in the same habitat. Future research can investigate the effect of brass in the SSF for metal removal in water, promoting the presence of genera such as *Lactobacillus*^50,51^.

## Conclusions

The evaluated SSF systems removed more than 90% of the coliforms, suggesting that they are a potential alternative for contaminated water treatment. The addition of copper, iron, and brass did not significantly improve their performance. Iron (H′ = 11.03) and copper (H′ = 10.5) did not modify *schmutzdecke* prokaryotic community in the SSF, but brass changed the prokaryotic diversity, favoring the population of *Streptophyta* and acetic acid genus species in addition to *Lactobacillus*, compared with all other SSF tested systems, resulting in an oligodynamic effect on the *Betaproteobacteria*, *Chloracidobacteria*, and *Cytophagia* classes.

## Supplementary information

Supplemenary material

## Data Availability

All data generated or analyzed during this study are included in this published article (and its Supplementary Information files).
