# Supplementary material for: Streptophyta and Acetic Acid Bacteria Succession Promoted by Brass in Slow Sand Filter System Schmutzdeckes
Source: Sci Rep. 2019 May 7;9:7021. doi: 10.1038/s41598-019-43489-9 (PMC6504865; doi:10.1038/s41598-019-43489-9)
Supplement: Supplementary file 1 — Supplemenary material [file 41598_2019_43489_MOESM1_ESM.docx]

## *Streptophyta* and Acetic Acid Bacteria Succession Promoted by Brass in Slow Sand Filter System *Schmutzdeckes*

Ma. Carmen E. Delgado-Gardea ^1,2,^ Patricia Tamez-Guerra^1^, Ricardo Gomez-Flores^1^, Mariela Garfio-Aguirre^1^, Beatriz A. Rocha-Gutiérrez^2^, César I. Romo-Sáenz^1^, Francisco Javier Zavala-Díaz de la Serna^2^, Gilberto Eroza-de la Vega^2^, Blanca Sánchez-Ramírez^2^, María del Carmen González-Horta^2^ and Rocío Infante-Ramírez*^2^

^1^Universidad Autónoma de Nuevo León, Facultad de Ciencias Biológicas, Departamento de Microbiología e Inmunología, Ave. Universidad s/n, San Nicolás de los Garza, N.L., México 66450. [carmen_060@hotmail.com](mailto:carmen_060@hotmail.com) (M.C.E.D.-G.); [patamez@hotmail.com](mailto:patamez@hotmail.com) (P.T.-G.); [rgomez60@hotmail.com](mailto:rgomez60@hotmail.com) (R.G.-F.); [aleiram_oifrag@hotmail.com](mailto:aleiram_oifrag@hotmail.com) (M.G.-A.); cesar_ivan_romo@hotmail.com (C.I.R.S.). ^2^Universidad Autónoma de Chihuahua, Laboratorio de Biotecnología, Facultad de Ciencias Químicas, Circuito Nuevo Campus Universitario No.1, Chihuahua 31125, México; brocha@uach.mx (B.A.R-G); fzavala@uach.mx (F.J.Z.-D.S.); [gerosa@uach.mx](mailto:gerosa@uach.mx) (G.E.-V.); [bsanche@uach.mx](mailto:bsanche@uach.mx) (B.S.-R.); carmengonzalez@uach.mx (M.C.G.-H.).

* Correspondence: [rir.infante@gmail.com](mailto:rir.infante@gmail.com) , Tel.: +52-(614)-236-6000 (ext. 4265).

**Supplementary material**


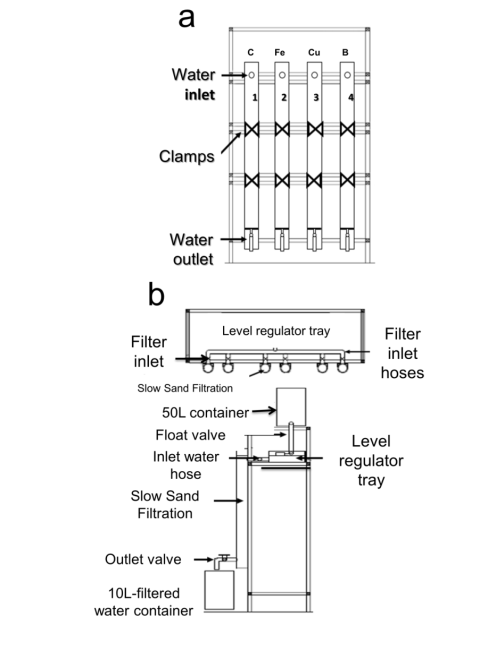


**Supplementary Figure S1.** Filtration system design. A) Front design of the filtration system where: C= control; Fe = iron; Cu = cooper; B = brass. B) Filtration system affluent and effluent specifications.

| Supplementary Table S1. Soil physicochemical characterization | |
| --- | --- |
| Analysis | **Result** |
| Texture | Sandy soil (A) |
| Apparent density | 1.41 g/mL |
| Real density | 2 g/mL |
| Porosity percentage | 30% |
| pH | 8.57 |

| Supplementary Table S2. Total sequence analysis | | |
| --- | --- | --- |
| Sample ID | High quality sequence | Sequence size |
| Control | 143,589 | 286 |
| Steel | 200,639 | 289 |
| Copper | 178,255 | 289 |
| Brass | 264,346 | 286 |
